# Is Renal Ischemic Preconditioning an Alternative to Ameliorate the Short- and Long-Term Consequences of Acute Kidney Injury?

**DOI:** 10.3390/ijms24098345

**Published:** 2023-05-06

**Authors:** Juan Antonio Ortega-Trejo, Norma A. Bobadilla

**Affiliations:** 1 Molecular Physiology Unit, Instituto de Investigaciones Biomédicas, Universidad Nacional Autónoma de México, Mexico City 04510, Mexico; 2 Department of Nephrology and Mineral Metabolism, Instituto Nacional de Ciencias Médicas y Nutrición Salvador Zubirán, Mexico City 14080, Mexico

**Keywords:** remote ischemic preconditioning (IPC), oxidative stress, inflammation

## Abstract

Acute kidney injury (AKI) is a global health problem and has recently been recognized as a risk factor for developing chronic kidney disease (CKD). Unfortunately, there are no effective treatments to reduce or prevent AKI, which results in high morbidity and mortality rates. Ischemic preconditioning (IPC) has emerged as a promising strategy to prevent, to the extent possible, renal tissue from AKI. Several studies have used this strategy, which involves short or long cycles of ischemia/reperfusion (IR) prior to a potential fatal ischemic injury. In most of these studies, IPC was effective at reducing renal damage. Since the first study that showed renoprotection due to IPC, several studies have focused on finding the best strategy to activate correctly and efficiently reparative mechanisms, generating different modalities with promising results. In addition, the studies performing remote IPC, by inducing an ischemic process in distant tissues before a renal IR, are also addressed. Here, we review in detail existing studies on IPC strategies for AKI pathophysiology and the proposed triggering mechanisms that have a positive impact on renal function and structure in animal models of AKI and in humans, as well as the prospects and challenges for its clinical application.

## 1. Generalities of Acute Kidney Injury

Acute kidney injury (AKI) is a very frequent syndrome that occurs in 10–15% of hospitalized patients, and its incidence is considerably higher in critically ill patients, which in turn, increases patient mortality [1,2]. Worldwide, it has been estimated that 13.3 million people per year have at least one episode of AKI, contributing to approximately 1.7 million deaths per year [3]. The most common cause of AKI is related to an ischemic process that induces a decrease in renal oxygenation with the subsequent generation of free radicals [4]. Several types of cells in the nephron are affected; however, the proximal tubular epithelial cells are the most compromised due to their dependence on aerobic oxidative metabolism [5]. Although epithelial cells have the capacity to activate reparative mechanisms, it has been reported that a maladaptive response may occur that leads to tubulointerstitial fibrosis; therefore, clinical and experimental studies have demonstrated that AKI is a risk factor for the development of chronic kidney disease (CKD) [6,7,8,9,10]. CKD is a global health concern since it has been estimated that 1.2 million people die annually due to this pathology [11]. Coupled with this, several studies have reported that 20.1% to 44% of AKI survivors are susceptible to suffering at least another AKI episode that increases both the progression to CKD and mortality, a situation that has already been termed recurrent AKI (rAKI) [12,13,14,15,16,17]. Interestingly and contradictory to clinical observations, experimental studies have shown that repeated and short ischemic insults [18,19,20,21,22,23,24,25,26,27,28,29,30,31,32], as well as two renal ischemia periods of the same magnitude, may induce renal protection, an effect that has been known as ischemic preconditioning (IPC) [33,34,35,36,37,38,39,40,41,42].

## 2. Pathophysiology of Acute Kidney Injury

AKI is characterized by a transitory loss of kidney function, limited to 7 days of duration, due to a decrease in renal blood flow (RBF). The main causes of AKI are sepsis, a decrease in intravascular volume, renal ischemia, and nephrotoxic drugs, among others. AKI is diagnosed when the serum creatinine is elevated by ≥0.3 mg/dL in 48 h, by an increase of ≥1.5 times the basal values, or by a decrease in the urinary output to <0.5 mL/kg/h during 6 h [43].

During an AKI episode, the hypoxic state damages the endothelial cells, which results in increased production of vasoconstrictor factors such as endothelin-1, prostaglandin H2, angiotensin II, and thromboxane A2. Simultaneously, there is an increase in adhesion molecules (ICAM-1 and/or β-integrin) [44] and in proinflammatory cytokines, such as TNF-α, MCP-1, IL-6, IL-32, IL-1β, IL-18, and TGF-β. These events allow immune cell infiltration and phagocytic cell activation, such as neutrophils and macrophages. Although necessary to clean cell debris and apoptotic cells, their recruitment and activation generate a local release of proinflammatory signals, reactive oxygen species (ROS), and proteases that perpetuate renal dysfunction and tissue injury [45]. As a result of vascular damage, there is a disruption of the extracellular matrix and an alteration of the cytoskeleton that promote modifications in cell–cell interactions, which in turn induce greater vascular permeability. If this inflammatory response is not regulated, it can lead to cell death, drastically reducing the number of vessels and worsening the hypoxic state, ultimately resulting in tubular interstitial fibrosis [46].

The damage to epithelial cells that occurs in AKI appears primarily in the S2 and S3 segments of the proximal tubule. An imbalance in the ATP supply disrupts the cytoskeletal architecture and β-actin and tubulin filaments are disrupted, inducing loss of the brush border, loss of cell polarization, and mislocalization of membrane proteins [47]. The disruption in the localization of transporters and adhesion molecules deregulates the absorption or secretion functions of these segments, as well as alter cell–cell interactions, causing a detachment of both viable and nonviable cells and increasing solute wasting and protein excretion in urine, which, in turn, may combine with the Tamm–Horsfall protein (THP) and fibronectin to form casts that obstruct the tubules, which are a hallmark of AKI [48]. The cells of the tubular epithelium have the ability to proliferate and replace the lost cells, thus, repairing the damaged epithelium [49,50]. If the whole process occurs correctly, the kidney functions are restored in a few days. However, in some cases, maladaptive mechanisms are activated, leading to a failure in kidney repair and favoring progressive injury. Some of these mechanisms are tubular cell arrest in the G2/M cycle [51], chronic inflammation and cell infiltration [52], myofibroblast production, and increased extracellular matrix deposition [53].

## 3. Long-Term Consequences of AKI

Experimental, epidemiological, and clinical studies have reported that AKI is an independent risk factor for the development of CKD, which has been termed AKI to CKD transition [9]. The course of this transition is determined by the initial insult severity and duration, where the age of the patients is another preponderant factor [54,55]. Many efforts have been made to understand the mechanisms that managed the AKI to CKD transition. The most relevant include chronic hypoxia [56], vascular rarefaction [57] proliferation of epithelial cells with excessive production of TGF-β [7], transdifferentiation of pericytes into myofibroblasts [58], and chronic stress of the endoplasmic reticulum [59]. Many other mechanisms, however, remain to be elucidated.

## 4. Ischemic Preconditioning (IPC)

In 1986, Murry et al. [60] described that four cycles of 5 min of ischemia in a dog’s left anterior descending (LAD) coronary artery induced protection against an insult of greater magnitude, i.e., 90 min of ischemia, which was evidenced by a reduction in 75% of the myocardial infarction area. Since then, this process has been called ischemic preconditioning (IPC), and it consists of making the tissue tolerant by performing repeated episodes of ischemia, alternated with reperfusion, before a sustained and more severe ischemic damage is produced.

Nevertheless, two years earlier Zager RA, et al. [41] showed in Sprague Dawley rats that a mild or severe bilateral renal ischemia (BRI) of 25 or 40 min, performed 18 or 48 h before a second hit of 40 min of BRI, produced renal protection, which was evidenced by a significant reduction in serum creatinine concentration (SCr) compared to the group with only one IR event. One year later, the same group demonstrated that a 15 min period of IR performed 3.5 to 24 h before a second insult of greater magnitude (25 min) was sufficient to provide renal protection; interestingly, when the interval was shortened to 0.5 h between each episode of IR, kidney protection was not achieved and even further damage resulted [42]. These results strongly suggest that the benefits of IPC require a longer period of time for the protective molecular mechanisms to become established.

Since then, continuous research has been carried out to determine the best model of IPC and its respective protection mechanism with the intention of being used in the clinical setting as a possible therapeutic intervention to reduce or avoid AKI.

## 5. Impact of One or More Short Cycles of Renal IPC on a Larger Ischemic Insult

It is well known that brief cycles of IR are enough to induce renal preconditioning. Table 1 presents some of the studies that evaluated the effect of one or more brief cycles of IR before a larger ischemic insult is performed. In the 15 studies reported, the effect of 1, 2, 3, or 4 short ischemic cycles, ranging from seconds to 15 min, were evaluated; the most common protocol used was 5 min of ischemia and 5 min of reperfusion. The second ischemic challenge ranged from 30 to 60 min, with half of the studies employing the experimental model of bilateral renal ischemia (BRI) and the other half the unilateral renal ischemia (UIR). As is appreciated in Table 1, there is no consensus on establishing effective renal protection depending on the ischemic period, the number of cycles, the interval between each cycle, or the experimental model used; still, most of these studies observed a clear renoprotection. A meta-analysis carried out by Wever et al. [61] showed that the IPC strategy is strongly influenced by the animal species used, which could explain some differences found in all these studies. For example, Joo et al. [19] and Mahfoudh-Boussaid et al. [24] found an increase in p-Akt, but Li et al. [27] showed a decrease, and in all these three studies, the outcome was the same, renoprotection. Khalid U. et al., compared multiple short cycles of IPC to one of moderate magnitude before a major insult is induced in rats, finding that 4 cycles of 2 min of ischemia separated by 5 min of reperfusion resulted in better renoprotection [28]. To our knowledge, the only study that did not find protection was done on Wistar rats, with IPC consisting of 3 cycles of 5 min of ischemia and 5 min of reperfusion [61]. Notably, most of these studies evaluated the IPC effects only 24 or 48 h after the preconditioning, except for Timsit et al. [23] and Zhang et al. [31], who evaluated the animals after 15 and 42 days, finding major survival of the animals that underwent IPC. Therefore, the impact of short periods of IPC on the long-term consequences of an AKI event remains to be fully defined.

## 6. Effect of Two or Three IR Episodes of Similar Severity

A way for evaluating the impact of the IPC upon a subsequent insult (hit) of similar magnitude has been extensively studied, inducing two BRI episodes of similar magnitude, ranging from 15 to 40 min, in both rats and mice. As shown in Table 2, it is more common to find two insults lasting 30 min and that were spaced from 0.5 h to 15 days. This table highlights the fact that in more than half of these studies, the interval between each IR was 7 days, which makes these studies more comparable. Furthermore, the animals were studied in most cases 24 to 48 h after the last insult. However, little is known about the long-term impact of the IPC. As summarized in Table 2, there was an improvement in renal function in almost all cases when compared to the damage induced by a single BRI episode, showing a window of protection when each ischemia is carried out between 3 to 8 days and even at intervals of 15 days. Only in the study by Dong, Y. et al. [62] was a worsening in renal function reported. The difference between this study and the rest is that the IR only occurred unilaterally, which suggests that different mechanisms are activated between BRI and URI. There are practically no studies that have carried out more than two episodes of renal ischemia and their long-term impact, except one that we recently published. In our study, AKI to CKD transition was evaluated after three mild (20 min) or three severe (45-min) episodes of IR (3IR) and compared with a single moderate or severe IR episode (1IR). The animals were followed for 9 months, and the 1IR group (20 or 45-min) developed CKD as evidenced by progressive proteinuria and renal fibrosis. Interestingly, the long-term consequences of AKI were markedly ameliorated in the 3IR group. Our study shows that renal preconditioning by three cycles of moderate and severe IR remarkably reduced the long-term consequences of AKI [63].

## 7. Remote Ischemic Preconditioning (rIPC)

The renoprotective effect of the IPC does not necessarily have to be generated in the tissue to be protected, but IPC could be performed in distant tissues, developing messengers that travel to different organs activating protective signaling pathways. This kind of precondition is known as remote IPC (rIPC) [64]. This phenomenon has been described first in the heart and brain, and shortly after in the kidney. The great advantage of the rIPC is its potential use in the clinical setting. As shown in Table 3, most of the experimental studies performed short periods of 5 min of ischemia on the hindlimb or femoral artery, and the challenges were mostly BRI of 45 min and evaluated 24 h later. All the studies reported renal function improvement except for the study performed by Kierulf-Lassen, C. et al. [65]. This protection was associated with an increase in the activity of antioxidant enzymes, such as SOD and catalase, preventing oxidative stress and death cell. A downregulation in cytokine levels and infiltration cells is also an important finding. The only two studies that evaluated the chronic effect of rIPC reported opposite results. In one study, there was an inhibition of TGF-β expression, whereas in the other hand, tubulo-interstitial fibrosis was found. This difference may be explained by the different species studied. Additionally, an attempt has been made to elucidate the molecules that could be responsible for exerting remote protection [66,67,68,69]. It has been reported that there is crosstalk between kidneys and various organs, such as intestine, heart, spleen, and brain [65,70,71]. Apparently, the molecules responsible for carrying the message to the kidneys may be the same regardless of which organ the preconditioning stimulus is applied to. Very few of the studies address this question, but the few that do report implied molecules, such as hormones, cytokines, and nitric oxide (NO). It seems that the renoprotective molecules involved must be in the circulation in order to reach distant sites, and one approach that could explain it is through exosomes.

## 8. Renoprotective Mechanisms Induced by Short and Long Cycles of IPC

Several mechanisms have been described as responsible for the renoprotection conferred by short or long cycles of IPC shown in Table 1, Table 2 and Table 3. Many of them converge in the different reported studies and are described below.

***Reduction of Oxidative Stress***: Although there are differences among species, there are conserved mechanisms that are activated by the IPC to protect cells from injury. It is well known that during AKI, there is a failure to produce ATP in proximal tubular cells due to mitochondrial damage. The mitochondria are the main source of reactive oxygen species (ROS) and if their levels are not controlled by antioxidative enzymes, such as catalase or superoxide dismutase (SOD), cells could suffer apoptosis or necrosis. It has been reported, however, that low levels of ROS are required for proper cell function by activation signaling pathways or protein modifications [77,78]. Until a certain point, ROS are also involved in the IPC, since there is an increase in superoxide, hydrogen peroxide, and lipid peroxidation that persisted 8 days later. All these events are accompanied by an increase in 33% of the total antioxidant capacity [25], an elevation in manganese superoxide dismutase (MnSOD) activity, and a decrease in angiotensin II [35,38]. The correct regulation of ROS limits oxidative stress preventing lipid peroxidation, as has been demonstrated by the decrease in malondialdehyde (MDA) levels after IPC [23,27]. The positive involvement of ROS during IPC is partially lost when antioxidant reagents such as MNTMPyP (manganese(III) tetrakis(1-methyl-4-pyridyl) porphyrin) or N-acetylcysteine were administered, while other proteins, such as inducible nitric-oxide synthase (iNOS) or heat shock protein of 25 kDa (HSP25), which are also overexpressed by IPC, were not affected [38,39]. The role of antioxidant enzymes activity seems to be crucial in the rIPC, since SOD and catalase activity increase when the rIPC is performed in the small intestine protecting the kidneys against ischemic injury [70]. It is well known that the decrease in ROS generation prevents apoptosis by inducing antiapoptotic proteins such as Bcl-2 and by reducing BAX and cleaved Caspase 3 levels [66,73]. This reduction of apoptosis could also be explained by the increase in the phosphorylation of Akt and ERK1/2 in the renal tissue of animals with rIPC performed in the heart [79].

***Nitric Oxide and pAkt Pathway Involvement:*** During AKI, the elevation of nitric oxide (NO) seems to be secondary to the enhanced iNOS activity, which is a vasodilator factor. Gene deletion of iNOS or its pharmacological inhibition with L-N6-(1-iminoethyl) lysine (L-NIL) increases kidney susceptibility to a second ischemic insult. Interestingly, the deletion of the eNOS gene has no effect on this susceptibility [39]. Although the protection does not disappear completely, iNOS is important to the late protection because of its sustained expression up to 12 weeks after performing IPC [19]. One of the proofs that demonstrates the importance of NOS enzymes was evidenced in eNOS-deficient mice, where there was no IPC protection [18] or when a nonselective NOS inhibitor, such as N-nitro-L-arginine methyl ester (L-NAME), was administrated, showing a partial loss of IPC protection [18,22,24]. Furthermore, it has been observed that the activity of iNOS progressively increases 24 h after ischemia injury and could explain its crucial role in long-term protection [19]. Medullary congestion and tubular necrosis are prevented by IPC, possibly due to the vasodilatory effects that NO has and the specific localization of eNOS (endothelium) and iNOS (glomerulus and proximal tubules), helping to maintain a better blood flow and reducing the hypoxic risk [22]. Besides inhibited NOS enzymes, a decrease in the stability of HIF-1α has been observed, preventing the expression of its target genes involved in adaptation to low oxygen levels [24], suggesting that NO also helps to stabilize HIF-1α [80].

As was reported before, the role of NO in kidney protection has already been demonstrated by IPC in local tissue but its participation in remote protection is controversial. The main difficulty in proposing NO as a mediator in rIPC is its short half-life, which is around 2 milliseconds or less [81], making it impossible for NO to travel through circulation to distant organs. However, the NO oxidation product, nitrite, which has a half-life of around 60 min [82] and has vasodilator and cytoprotector effects, could be the mediator of NO generated by the rIPC [83]. Regardless of whether NO or nitrite is responsible for the renoprotective effects, Gholampour et al. [67] showed that when rIPC is performed in the left femoral artery, the renoprotection observed after inducing BIR was associated with decreased lipid peroxidation and increased GPX and catalase activity. This antioxidant effect was diminished when L-NAME was administrated, indicating the participation of NO in the renoprotection observed.

Another mechanism involved in the IPC is the participation of Akt and its phosphorylation (p-Akt), which is a serine/threonine kinase that participates in cell survival [24]. The IPC protection has been associated with the activation of the Akt signal pathway, which, in turn, induces antiapoptotic proteins (Bcl-2 and Bcl-x) and prevents DNA fragmentation [36]. Nevertheless, Akt and p-Akt involvement in the IPC seems to depend on the species and the strain studied. While in mice C57BL/6 and Wistar rats, IPC promotes elevation in p-Akt, an effect that is associated with cytoprotection by counteracting apoptosis [19,24], in Sprague Dawley rats, IPC reduces the activation of the Akt pathway, preventing the phosphorylation of NF-κB, a master transcription factor that regulates inflammatory responses; therefore, IPC was associated with a reduction in renal inflammation [27]. These differences may be explained through the particular signal pathway activated for the IPC. On the one hand, it has been found that the protection is through activating the phosphatidylinositol-3 kinase (PI3K)-Akt pathway or through protein kinase C (PKC) in mice [19], and, on the other hand, the overexpression of HO-1, induced by the master transcription factor Nrf2, was observed in rats after the IPC [27].

***Heat Shock Proteins Induction:*** Other mediators involved in the IPC are the heat shock proteins (HSPs) that participate in several cellular functions, such as the correct protein folding, intracellular protein transport, translocation of transcription factors, regulation of cell signaling in inflammation, apoptosis, and proliferation [84]. One of the hallmarks of IR inducing renal injury is the loss of epithelial cell polarization, where HSP25 plays an important role in stabilizing actin microfilaments [85]. In this regard, it has been reported that the induction of HSP25 is dependent on the ischemic intervals; however, there is a greater HSP25 overexpression after the IPC, most likely to counteract the renal damage [38,39,40]. Another HSP involved in the IPC protection is HSP32, also known as heme-oxygenase-1 (HO-1) [27]. Indeed, we demonstrated that 24 h after the third round of ischemic insults with 10-day intervals, there was a significant increment in HO-1 and M2 macrophages, together with an anti-inflammatory response mediated by a decrease in NF-κB-p65 phosphorylation and IL-6. Thus, repeated episodes of IR with 10-day intervals induced long-term renal protection accompanied with HO-1 overexpression and an increase in M2 macrophages [63].

Furthermore, when there is an accumulation of unfolded protein in the endoplasmic reticulum (ER), the unfolded protein response (UPR) is activated. One principal ER chaperone is the 78-kDa glucose-regulated protein (GRP78), also known as binding immunoglobulin protein (BiP), which facilitates the correct folding of proteins and helps to translocate new synthetized peptides into the ER membrane [86]. Interestingly, the IPC has been reported to increase the expression of GRP78/BiP and of proteins, such as tumor necrosis factor receptor-associated factor 2 (TRAF2) and activating transcription factor 4 (ATF4), which are involved in decreasing ER stress-induced apoptosis [24].

***Amelioration of Renal Inflammation:*** It has been reported that the IPC reduced the phosphorylation of mitogen-activated protein kinase 7, 4, and 3/6 (MKK7, MKK4 and MKK3/6, respectively), inhibiting the downstream activation of JNK and p38, which are involved in the induction of adhesion molecules in endothelial cells and proinflammatory cytokines [40]. Several studies have shown that the IPC reduces proinflammatory cytokine expression, including that of TNF-α, IL-6, IL-1β, IL-17, and MCP-1 [25,30,31], as well as downregulation of the TLR4/NF-κB pathway that limits the infiltration of immune cells into the kidney [27,30,32]. Zhang et al. [32] found that the IPC does not seem to prevent immune cell infiltration into the kidney, but inhibits dendritic cell maturation. Splenocytes from animals with IPC had more Tregs and mature CD11c+ macrophages/dendritic cells (DC) mediating immune tolerance by reducing cytokine-secretory responses, including TNF-α, IFN, MCP-1, and IL-6 [34]. Interestingly, this IPC protective effect could be translated to T cell-deficient mice, when Tregs from mice with IPC were transferred [33]. Cho et al. described that the protection conferred by this transfer strategy is mediated by Treg cells [34]. The role of macrophage infiltration in the IPC has also been studied. In BALB/c and C57BL/6 mice, the administration of lipo-clodronate (which depletes macrophages) that reduced the amount of macrophages did not modify the protective effect of IPC [34,37].

Similarly, systemic proinflammatory cytokines, such as IL-1 and IL-6, are reduced after an rIPC, which may decrease immune cell infiltration and pro-apoptotic proteins in remote organs [66,69,71,74,79]. In addition to this, the NF-κB is also inhibited [63,69,71,73], provoking an anti-inflammatory response accompanied by an elevation of IL-10 as an anti-inflammatory cytokine [71]. It has also been reported that the elevation of serum TNF-α promotes renalase expression. Renalase is an amino oxidase that originates in the kidney that can regulate blood pressure and serve as a prosurvival/growth factor [87,88]. Wang et al. demonstrated that renalase is required for kidney protection induced by rIPC, since when a renalase siRNA is administrated, the protection is abolished [66]. TGF-β and its downstream signaling proteins Smad2 and Smad3 are well-known mediators of renal fibrosis in the long consequences of IR [7]. Nevertheless, rIPC avoids the induction of this cytokine with the concomitant decrease in fibronectin, collagen I and III, and α-SMA, and the consequent renal fibrosis [75]. This protection seems to depend on the species and the type of rIPC location, because when rIPC is applied in the limbs of rats, there is no renal fibrosis after three months [75], but when the rIPC is located in the heart of mice, renal fibrosis is worse after four weeks [76].

***Involvement of Autophagy in the protection conferred by IPC:*** Autophagy regulates the renewal and death of senescent or damaged cells. There are three different types of autophagy: macroautophagy, microautophagy, and chaperone-mediated autophagy. In general, it has been reported that autophagy-related proteins (ATG) are induced by IPC [89]. Beclin-1, a Bcl-2 interacting protein, is the main upstream regulator of autophagy and served as a scaffold to Vps34, Vps15, and ATG14L, which participate with the vesicle nucleation [90]. In animals undergoing IPC, the induction of Beclin-1 occurs as fast as 6 h post-IR and remains elevated for 24 h [26].

The second step of autophagy is the autophagosome formation ATG12-conjugation system, the increase in microtubule-associated protein 1 light chain 3 (LC3), and phosphatidylethanolamine (LC3-II) expression. These proteins are responsible for elongation and closure of the phagophore membrane, which could be derived from mitochondria, endoplasmic reticulum (ER), Golgi, or plasma membranes. It has been shown that after IPC, the increase in LC3-II and Atg12 promoted autophagy and blocked apoptosis in the kidney [20,27,31]. Moreover, the concentration of LC3-II on the apical side of the proximal tubule cells in animals with IPC has been observed [20]. IPC not only induced LC3-II accumulation but also the maturation of autolysosomes, the final step for the clearance of autophagic cargo. This specific accumulation of autophagosomes on the apical side of proximal tubule cells could be due to the enrichment of ATP-dependent transporters, generating an elevated rate of mitochondria replacement. 

The correct and regulated form of damaged mitochondria degradation, known as mitophagy, is another mechanism of the IPC protective effect [20,21,25]. This is mediated by the induction of PINK1 (PTEN induces putative kinase-1) that surrounds damaged mitochondria and, therefore, serves as a sensor, and BNIP3L (BCL2-interacting protein 3-like), and FUNDC1 (FUN14 domain containing 1) that are receptors that initiate mitophagy under hypoxic conditions and interact with UNC-51-like kinase 1 (Ulk1). After IPC, PINK1 was activated, but when its expression was reduced with a shRNA, the mitolysosomes were almost blocked, along with the protection [20]. Interestingly, in renal proximal tubule-conditional Fundc1-deficient mice, the LC3II expression is downregulated together with loss of IPC protection [20,21].

***Post-translational modifications involved in the IPC protection.*** Interestingly, protein acetylation is higher in the renal tissue of aging rats, which results in the abolishment of the IPC protective effect [25]. microRNAs (miRNA) regulate gene expression, not only maintaining cellular functions but also regulating responses to tissue injury. miRNAs bind to mRNAs and inhibit or promote translation. There are some microRNA signatures established during the IPC. Yamamoto. et al. found that some miRNAs, such as, miR-17-3p and miR-19s, inhibit PTEN, whereas miR-34a activates it [29]. Interestingly, microRNAs such as miR-21-5p, miR-22-3p, and miR-222-3p that are involved in fibrosis, angiogenesis, and vascular remodeling are suppressed by IPC [28].

***Hormones participation in the IPC.*** The key molecule responsible for the communication between the brain and the kidney is erythropoietin (EPO) [68]. EPO is a glycoprotein produced in the kidney whose main function is to stimulate the production of red blood cells, but it has also been proposed as a protective hormone against ischemic brain injury [91]. Another protective effect is mediated by the heteromeric complex known as the tissue protective receptor (TPR), a complex made up of the EPO receptor (EPOR) and the common β receptor (βCR), a subunit shared by type 1 cytokines [92]. TPR could activate several signaling pathways, one of which involves PI3K and Akt. Activation of this pathway promotes the phosphorylation of glycogen synthase kinase 3β (GSK3β), inhibiting the mitochondrial permeability transition (MPT) and stabilizing mitochondrial function [68,93]. The heart also has close communication with the kidney, as is the case of rIPC by permanent ligation of the LAD artery, in which chronic hypoxia protects the kidney against UIR. These animals exhibit lower levels of BUN and Kim-1, accompanied by increased EPO, PDK1, glut1, and VEGF mRNA levels. However, these results should be taken with caution, since this protection was not observed 4 weeks after the UIR; instead, worse renal fibrosis was exhibited [76].

***Alteration in cellular metabolism.*** IR injury provokes inflammation, osmotic dysregulation, and energy metabolism perturbation. Some differentially produced metabolites, such as ceramide, acylcarnitine, betaine aldehyde, adenosine, and glucosylceramide, could explain rIPC protection because of their involvement in these pathways [72,93]. Other relevant consequences could be post-translational modifications, as is the increase in protein O-GlcNAcylation, a form of glycan union with proteins due to rIPC. This process has been reported to regulate HSP40 and HSP70 and CHOP expression and attenuate NF-κB activity [73].

***Exosomes as a transport mechanism in the rIPC.*** Exosomes could be a crucial mediator in the renoprotection generated by distant IPC, reaching the damaged tissue from their origin in remote tissue. These microvesicles contain a variety of components, such as protein, RNA, lipids, and enzymes, conferring a role in various cellular processes such as, regulation of gene transcription and translation, inflammation regulation, and cellular communication [94,95]. Pan T et al. [69] demonstrated that in the rIPC, the increased generation of exosomes could reach several organs including the kidneys, which was associated with a reduction in serum creatinine and urinary NGAL. These exosomes contain MiR-21, an antiapoptotic miRNA. Another important finding was that MiR-21 has also been found in serum exosomes from human with rIPC. The exosome content determines the trigger mechanisms and the effects in distant tissues, mainly provoking an anti-inflammatory response and inhibiting NF-κB activity [69].

## 9. The Remote IPC in the Clinical Setting

AKI is one of the pathologies in which the applicability of rIPC has been proven to be effective by reducing its frequency and severity in patients susceptible to it. Because of rIPC advantages and low invasiveness, several clinical trials have been conducted to evaluate its efficacy and safety. Although some studies report promising results, the clinical utility of rIPC to prevent AKI is still unclear. As it can be appreciated in Table 4, the protocol used in most of the studies using rIPC is practically the same, which involves 3 to 4 cycles of 5 min of arm ischemia, which was achieved through arm compression with a cuff inflated mostly to 200 mmHg. Even though the same protocol was followed in several studies, the results are contrasting, as we will see below.

In 2007, Ali, ZA et al. [96] included 82 patients in a study on repairing abdominal aortic aneurysms (AAA) who were randomized into two groups: one received rIPC through two cycles of intermittent cross-clamping of the common iliac artery for 10 min, followed by a same period of reperfusion, and the other was treated without rIPC. Interestingly, rIPC reduced the incidence of postoperative myocardial injury (27% that can be caused by myocardial infarction), myocardial infarction (22%), and renal impairment (23%). In another study that included 40 AAA patients with rIPC in both lower limbs, the urine albumin/creatinine ratio decreased (46%) in comparison with the control group without rIPC [98]. Most clinical trials seeking to reduce AKI have studied rIPC in patients who have undergone cardiac surgery. Venugopal et al. [102] reported a decrease in AKI incidence by 14.5% when rIPC was applied to 78 patients. Deftereos et al. [97] included 220 patients undergoing cardiac surgery in a study and found that AKI incidence was 29.5% in the control group (*n* = 109), whereas it was reduced to 12.4% in the rIPC group (*n* = 111). In a similar study, which included 160 patients under cardiac surgery, the AKI incidence was reduced from 47.5% to 30% in patients with rIPC compared to controls [113]. Zhou, H et al. [114] found that the postoperative AKI incidence of 73.8% in 65 patients decreased to 55.4% in 65 patients with rIPC. Another study involving 28 patients found that in one-half of them with rIPC, there was a 64% decrease in cardiac surgery associated with AKI [108]. Interestingly, Zarbock et al. [105] evaluated the short- and long-term effects of rIPC in 240 patients undergoing cardiac surgery. Half of them received the rIPC strategy, finding not only that AKI cases were significantly reduced in the first 72 h, but also the incidence of long-term adverse renal events was reported after 90 days, such as all-cause of mortality, renal replacement therapy, and persistent renal dysfunction without dialysis [107]. Furthermore, different periods of rIPC were studied in 100 patients subjected to cardiac surgery. Intriguingly, all periods of rIPC studied were associated with an increase in tissue inhibitor of metalloproteinases (TIMP-2) and insulin-like growth factor-binding protein (IGFBP7), which in turn correlated with a reduction in AKI cases [110].

The rIPC has also been evaluated in 178 patients undergoing coronary artery bypass graft (CABG); it was found that AKI incidence decreased by 48% in the rIPC group compared to the control group [101]. In addition, the reduction in renal injury biomarkers, such as cystatin C and NGAL, has been reported after rIPC in 60 patients with CABG [100].

Patients undergoing coronary artery angiography (CAA) are a population at risk of developing contrast-induced AKI (CI-AKI); therefore, several clinical trials have studied whether rIPC could be used as a renal protective intervention. Er et al. [115] randomized 82 patients, half of whom served as controls and the other half received rIPC, finding a decrease in CI-AKI incidence from 40% to 12%, respectively. Savaj, et al. [104] studied 96 diabetic patients who underwent CAA and who were randomly divided equally into the control and rIPC group; in spite of an observed reduction of SCr due to rIPC, the CI-AKI incidence was not different between groups. These results suggest that the heterogeneity of the patients studied prevented seeing a difference in the incidence of AKI. In another study, the benefit of rIPC was evaluated in 60 patients with pre-established CKD and who underwent angiography, half of them without rIPC and the other half with rIPC; a decrease in the incidence of CI-AKI was observed from 26.9% to 7.7%, respectively [111]. Similar results have been reported by Yamanaka et al. [106], Zagidullin et al., [109] Moretti et al. [112], and Elserafy et al., finding a decrease in the incidence of CI-AKI [116]. Additionally, Thielman et al. [103] and Zimmerman et al. [99] studied patients under CABG or valve surgery and observed a SCr decrease due to rIPC of 3 cycles of 5 min of ischemia in the upper arm or lower extremity, respectively.

In addition to these clinical studies that clearly demonstrate that rIPC produces renoprotection, there are a number of studies that report that the rIPC strategy in patients with risk of developing AKI does not exert renal protection or influence the incidence of AKI [117,122,123,124,126,128,129,130,131,132], particularly in CI-AKI [64,125,127,133,135,136] or on renal injury biomarkers [118,119,120,121]. These discrepancies could be due to several factors that reflect the complexity of the clinical trial design and the influence of comorbidities of each studied population. All these variables that impact the response to rIPC were reviewed by McCafferty K et al. [137] in animal models and other organs besides the kidney. In these studies, the included groups were composed of young and healthy animals. Unfortunately, in most of the clinical trials, these conditions do not occur because the patients evaluated exhibit one or more comorbidities, and some of them are elderly. Undoubtedly, more studies are needed to consider these factors and to determinate the influence of each one on the possible renoprotection by rIPC.

Considering the differences in the clinical trials mentioned above, it is crucial to deepen our knowledge of all the possible molecular and cellular mechanisms involved; however, it is necessary to carry out clinical trials designed to assess the real impact of rIPC, pondering the specific influence of comorbidities in the studied population.

**Figure 1:**
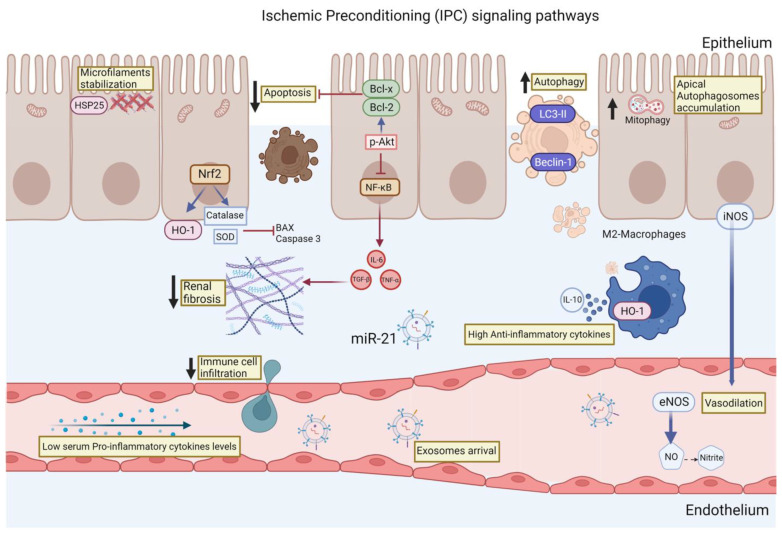
**Main renoprotective signaling pathways induced by the ischemic preconditioning (IPC):** (1) The induction of HSP27 allows the stabilization of the microfilament, which blocks epithelial cells from losing their polarization, a classic alteration observed after an IR episode. (2) Increased translocation of the transcription factor Nrf2 to the nucleus, where it induces the transcription of antioxidant enzymes such as catalase, superoxide dismutase (SOD), and heme-oxygenase (HO-1), which prevents the formation of reactive oxygen species (ROS). (3) Inhibition of epithelial cell apoptosis by reducing ROS and increasing the expression of antiapoptotic proteins such as Bcl-x and Bcl-2. (4) Although autophagy, particularly mitophagy, is increased, this allows for better regulation of damaged mitochondria. (5) Decreased activation of the proinflammatory transcription factor NF-κB, which also decreases the release of proinflammatory cytokines such as IL-6 and TNF-α, which, in turn, prevent the generation of renal fibrosis in the long term. (6) Increase of cells with an anti-inflammatory profile such as M2-macropaghes that remove cell debris and secrete anti-inflammatory cytokines such as IL-10. (7) Finally, some molecules such as NO, nitrites, and exosomes seem to be involved in the remote actions of IPC.

Finally, the signaling pathways described so far that participate in the renoprotection conferred by the IPC are summarized in Figure 1. These pathways included actin microfilament stabilization, autophagy induction, and reduction of oxidative stress through the induction of antioxidant enzymes such catalase and SOD. The resulting decrease in the generation of free radicals leads to the inhibition of apoptosis, and, in particular, to the turnover of mitochondria through mitophagy. Coupled with this, the IPC also influences the inflammatory response that is activated during AKI by maintaining an anti-inflammatory profile, which in turn allows controlled removal of damaged cells without activating immune cell infiltration signals and without the production of extracellular matrix proteins. We believe that the knowledge of the signaling pathways involved during the IPC will allow the creation of new proposals for therapies that activate or regulate some of these signaling pathways, without forgetting the great potential that the rIPC has, above all because it is a simple, noninvasive, safe, and low-cost strategy.

## Figures and Tables

**Table 1:** Effect of one or more short cycles of renal ischemic preconditioning on a larger ischemic insult.

Study	Species	Protocol of IPC and Ischemic Injury	Study Period	Main Outcomes
**Yamasowa, H. et al. (2005)** [18]	C57BL/6J	3 cycles: 2 min of I and 5 min of R **Challenge: UIR 45 min**	6–48 h	**RF Improvement** ↓ necrosis and medullary congestion. eNOS-deficient animals were not protected.
**Joo, J.D. et al. (2006)** [19]	C57BL/6J	4 cycles: 5 min of I and 5 min of R **Challenge: UIR of 30 min**	1–24 h	**RF Improvement** ↑ ERK, Akt, and Hsp27 phosphorylation. ↑ iNOS.
**Choi, H.S. et al. (2017)** [30]	C57BL/6J	One cycle of 3, 5 or 7 min of I and 10 min of R **Challenge: URI 30 min (Nx in right kidney).**	24 h	**RF Improvement** ↓ Tubular damaged. ↑ Bcl-2 expression. ↓ Casp3. ↓ TNF-α, IL-6, IL-1β, MCP-1, TLR4, and NF-κB.
**Zhang, T. et al. (2018)** [32]	C57BL/6	One cycle of 15 min of I, 4 days before **Challenge: BRI of 30 min**	24 h	**RF Improvement** ↓ Necrosis, tubule dilatation and cast ↓ CD11^c+^ cells. ↑ Increase of dendritic cells
**Livinston, M.J. et al. (2019)** [20]	C57BL/6	One cycle of I and 1 h of R, then: **Challenge: BRI of 27 min**	48 h	**RF Improvement** ↓ Apoptosis ↑ mitophagy in proximal tubule PINK1, BNIP3L/NIX accumulation. ↑ FUNDC1. Promotes mitophagosome to mitolysosomes.
**Wang, J. et al. (2020)** [21]	C57BL/6	One cycle of 15 min of I and 1 h of R **Challenge: BRI of 30 min**	24 h	**RF Improvement** ↑ p-Fundc1 in Serine 17 ↓ Drp1 and apoptosis. Mitophagy activity stabilize.
**Zhang, S. et al. (2022)** [31]	C57BL/7	One cycle of 15 min of I, 4 days before **Challenge: BRI of 30 min.**	48 h and 42 d	**RF Improvement** ↓ Kim-1 and tubular damaged ↑ Survival along 4 weeks ↑ LC3II and ↓ p62 ↑ Bcl-2
**Chen, H. et al. (2008)** [22]	Wistar	4 cycles: 8 min of I and 5 min of R **Challenge: UIR of 45 min**	24 h	**RF Improvement** ↑ NO production ↑ GSH, SOD, GSH-Px expression and ↓ MDA. ↑ iNOS and eNOS expression
**Timsit, M.O. et al. (2008)** [23]	Sprague-Dawley	3 cycles: 5 min of I, and 5 min of R **Challenge: IR 60 min**	15 d	**RF Improvement** ↓ MDA and α-SMA.
**Mahfoudh-Boussaid, A. et al. (2012)** [24]	Wistar	2 cycles: 5 min of I and 5 min of R **Challenge: BRI of 60 min**	2 h	**RF Improvement** Better cell integrity and ↓ peroxidation. ↑ p-Akt, eNOS and HIF-1α expression. ↑ BiP protein levels. ↓ p-ERK, ATF4 and TRAF2
**Jankauskas, S.S. et al. (2017)** [25]	Wistar and OXYS	4 cycles: 15 s of ischemia and 15 s of R **Challenge: UIR of 40 min**	48 h	**RF Improvement** ↑ Antioxidant capacity. Avoids protein acetylation increase. ↓ Autophagy.
**Arantes, V.M. et al. (2018)** [61]	Wistar	3 cycles: 5 min of I and 5 min of R **Challenge: IR 30 min.**	24 h	No protection
**Xie, Y. et al. (2018)** [26]	Sprague-Dawley	4 cycles: 8 min of I, and 5 min of R **Challenge: UIR of 40 min**	24 h	**RF Improvement** ↑ Beclin-1, SGK-1, and p-SGK-1 ↓ Casp3. ↑ HIF-1α, EPO, HO-1, and Bnip3. Induce autophagy
**Li, J.R. et al. (2019)** [27]	Sprague Dawley	3 cycles: 2 min of I and 5 min of R, 15 min before **Challenge: BRI of 45 min.**	24 h	**RF Improvement** ↓ Kim-1 ↑ Nrf2 and HO-1 expression. ↓ Bowman capsule dilatation, cellular debris ↓ Interstitial CD68 infiltration ↓ MDA, p-Akt, and NF-κB activation. ↓ apoptosis, but increase autophagy
**Khalid, U. et al. (2021)** [28]	Lewis	One cycle: 10, 15 or 20 min of I and 20 min of R or 3 cycles: 2, 5 or 10 min of I and 5 min of R **Challenge: BRI of 45 min.**	48 h	**RF Improvement** ↓ NGAL, and Kim-1 ↓ miR-21, miR-221 and miR-222

RF = Renal Function, URI = Unilateral Renal Function, BRI = bilateral renal ischemia, Nx = nephrectomy, eNOS = endothelial nitric oxide synthetase, HIF = hypoxia inducible factor, ATF4 = activating transcription factor 4, TRAF2 = TNF receptor associated factor, SGK = serum/glucocorticoid-regulated kinase, EPO = erythropoietin, HO-1 = heme-oxygenase-1, Nrf2 = nuclear factor erythroid 2-related factor 2, MDA = malondialdehyde, NF-kB = nuclear factor kappa-light-chain-enhancer of activated B cells, NGAL = neutrophil gelatinase-associated lipocalin, Kim-1 = kidney injury molecule 1.

**Table 2:** Impact of one or more cycles of IPC of similar magnitude.

	Species	First Hit	Second Hit	Third Hit	Interval between Hits	RF Evaluation after Last Insult	Main Outcomes
**Zager, R.A. et al. (1984)** [41]	Sprague-Dawley rats	40 min of BRI	35 or 40 min of BRI	X	18 and 48 h	Until 5 d	**RF Improvement**
**Zager, R.A. et al. (1985)** [42]	Sprague-Dawley rats	15 min of BRI	25 min of BRI	X	0.5, 2.5 and 24 h	24 and 48 h	**RF Improvement**
**Park, K.M. et al. (2001)** [40]	BALB/c mice	30 min of BRI	35 min of BRI	X	8 and 15 days	24 and 48 h	**RF Improvement** Inhibition of JNK and p38 ↑ Hsp27 and Hsp72
**Park, K.M. et al. (2003)** [39]	BALB/c mice	15, 25, or 30 min of BRI	30 min of BIR	X	3, 6, or 12 w	24 h	**RF Improvement** ↓ MPO activity ↑ eNOS and iNOS
**Burne-Taney, M.J. et al. (2006)** [33]	C57/Bl6 mice	30 min of BRI	30 min of BRI	X	3 days	24 and 72 h	**RF Improvement**
**Jang, H.S. et al. (2008)** [37]	BALB/c mice	30 min of BRI	30 min of BRI	X	7 days	24 h	**RF Improvement** ↓ MPO activity ↓ Neutrophils infiltration
**Kim, J. et al. (2010)** [38]	C57/Bl6 mice	30 min of BRI	30 min of BRI	X	7 days	24 h	**RF Improvement** ↑ MnSOD activity ↑ HSP25 and iNOS
**Cho, W.Y. et al. (2010)** [34]	C57/Bl6 mice	30 min of BRI	30 min of BRI	X	7 days	24 h	**RF Improvement** ↓ Apoptosis ↓ Immune cells infiltration ↓ IL-6, TNF-α, IFN and MCP-1 ↑ Tregs CD4^+^, Foxp3^+^
**Jang, H.S. et al. (2012)** [36]	BALB/c mice	30 min of BRI	30 min of BRI	X	7 days	24 h	**RF Improvement** ↓ leucocyte infiltration ↓ cytoskeleton disruption ↓ DNA fragmentation ↓ Dead cells ↑ p-Akt ↑ antiapoptotic proteins
**Jang, H.S. et al. (2014)** [35]	C57/Bl6 mice	30 min of BRI	30 min of BRI	X	7 days	24 h	**RF Improvement** ↓ Tubular damage ↓ Ang II levels ↓ MDA ↓ Nox2, Nox4 and nitrotyrosine. ↑ Interstitial AT1R expression.
**Dong, Y. et al. (2019)** [62]	C57/Bl6 mice	30 min of URI	30 min of URI	X	7 days	7 days	**Worse RF** ↑ Renal fibrosis ↑ α -SMA, Collagen I ↑ Apoptosis ↑ Macrophages infiltration.
**Ortega-Trejo, J.A. et. al. (2022)** [63]	Wistar Rats	20 or 45 min of BRI	20 or 45 min of BRI	20 or 45 min of BRI	10 days	24 h and 9 months	**RF Improvement** ↓ Renal fibrosis ↓ Oxidative stress ↓ Tubular damage ↑ HO-1 ↑ M2 macrophages

RF = Renal Function, URI = Unilateral Renal Function, BRI = bilateral renal ischemia. X = Not performed. eNOS = endothelial nitric oxide synthetase, Hsp27 = heat shock protein of 27 kDa, Hsp72 = heat shock protein of 72 kDa, MPO = myeloperoxidase, MnSOD = manganese superoxide dismutase, iNOS = inducible nitric oxide synthetase, IL-6 = interleukin, TNF-α = tumor necrosis factor alpha, IFN = interferon, MCP-1 = monocyte chemoattractant protein-1, Foxp3 = forkhead box p3, NOx2 = NADPH oxidase 2, Nox4 = NADPH oxidase 4, AT1R = angiotensin II receptor type 1, SMA = smooth muscle actin, HIF = hipoxia inducible factor, HO-1 = heme-oxygenase-1, MDA = malondialdehyde, NF-κB = nuclear factor kappa-light-chain-enhancer of activated B cells.

**Table 3:** Significance of remote IPC in distant organs on the injury induced by IR.

Study	Species	Remote IPC Tissue	Challenge For Renal Ischemia	Time after rIPC	Main Outcomes
**Song, T. et al. (2007)** [70]	Wistar	3 cycles: 8 min of I and 5 min of R in **mesenteric artery** (2-weeks before, the challenge)	**UIR of 45 min**	24 h	**RF Improvement** ↓ tubular degeneration and necrosis ↓ MDA ↑ SOD and catalase activity
**Kierulf-Lassen, C. et al. (2015)** [65]	Wistar	4 cycles: 5 min of I and 5m of R in **Aorta**	**BRI of 37 min**	7d	No protection due rIPC
**Menting, T.P. et al. (2017)** [64]	Sprague-Dawley	3 cycles: 5 min of I and 5 min of R in Both **thighs**	**UIR of 30 min**	2 d	**RF Improvement**
**Cho, K. et al. (2017)** [72]	C57BL/6	3 cycles: 5 min of I and 5 min of R in the **Lower limb** 2 or 24 h before the challenge	**UIR of 45 min**	24 h	↓ histological damaged Early GPX1 induction Adenosine deaminase (ADA), purine nucleoside phosphorylase (PNP) and choline dehydrogenase (CHDH) decrease
**Shen, Y. et al. (2018)** [71]	Sprague-Dawley	3 cycles: 5 min of I and 5 min of R in the **Spleen**	**UIR of 45 min**	24 h	**RF Improvement** ↓ necrosis, tubule dilatation and infiltration ↓ serum TNF-α and IL-6 levels ↓ IKK-β and NF-κB p65 Increase of IL-10
**Gholampour, F. et al. (2018)** [67]	Sprague-Dawley	2 cycles: 2 min of I and 3 min of R in the **Left femoral**	**BRI of 45 min**	24 h	rIPerC improves CCr ↓ MDA ↑ GPX and catalase activity ↓ tubule and glomerular damaged
**Hu, J. et al. (2018)** [73]	Sprague Dawley	4 cycles: 5 min of I and 5 min of R in the **Right femoral artery** 6 weeks before the challenge	Nx 5/6	72 h	**RF Improvement** NGAL ↓ death cell ↓ BAX and Casp3. ↑ SOD activity ↑ O-GlcNAcilation
**Varga, G. et al. (2020)** [74]	Wistar rats	3 cycles: 10 min of I and 10 min of R in the **Right hind limb** 1 or 24 h before the challenge	**UIR of 45 min**	2 h	rIPC-1 increase mean arterial pressure rIPC-24 increase RBC and hematocrit White blood cell counts are stable in rIPC groups Moderate necrosis ↓ brush border gaps
**Zhou, C. et al. (2020)** [75]	Sprague-Dawley	3 cycles: 5 min of I and 5 min of R in the **Limb** each every day	**UIR of 45 min**	3 months	Inhibition of TGF-β expression
**Terker, A.S. et al. (2021)** [76]	C57BL/6	Permanent ligation of Left anterior descending coronary artery 9 weeks before the challenge	**UIR of 30 min**	4 weeks	**RF Improvement** No mortality ↓ renal oxygen tension ↑ HIF target genes: EPO, Slc2a1 and Pdk1 ↑ Hk1, Hk2, Pkm1, Pgm1 and gapdh. Develop worse fibrosis

RF = Renal Function, rIPC = remote ischemic preconditioning, URI = Unilateral Renal Ischemia, BRI = bilateral renal ischemia, Nx = nephrectomy, LAD = left anterior descending artery, MDA = malondialdehyde, SOD = superoxide dismutase, GPX1 = glutathione peroxides 1, IKK-β = inhibitor of nuclear factor kappa-B kinase subunit beta, TNF-α = tumor necrosis factor alpha, IL-6 = interleukin, IL-10 interleukin 10, NF-κB = nuclear factor kappa-light-chain-enhancer of activated B cells, NGAL = neutrophil gelatinase-associated lipocalin, BAX = Bcl-2 associated X-protein, HIF = hypoxic inducible factor, EPO = erythropoietin, Slc2a1 = solute carrier family 2 member 1, Pdk1 = phosphoinositide-dependent kinase 1, Hk1 = hexokinase 1, Hk2, hexokinase 2, Pgm = phosphohlucomutase, gapdh = glyceraldehyde 3-phosphate.

**Table 4:** Results Generated by Clinical trials with remote IPC on AKI incidence.

Study	Patients	IPC Tissue	Ischemic rIPC Protocol	Main Outcomes after rIPC
**RENOPROTECTION**				
**Ali, Z.A. et al., 2007** [96]	*n* = 82 (rIPC = 41)	Iliac artery	2 cycles of cross-clamping iliac artery for 10 min	↓ in renal impairment
**Deftereos, S. et al., 2013** [97]	*n* = 220 (rIPC = 111)	Artery	4 cycles of 30 s of ischemia with inflation of the stent balloon to the nominal pressure	↓ AKI incidence
**Walsh, S.R. et al., 2009** [98]	*n* = 40 (rIPC = 18)	Both lower limb	2 cycles of 10 min with cuff inflation until there was no audible Doppler signal in each leg	↓ albuminuria
**Zimmerman, R.F. et al., 2011** [99] **Yildirim, F. et al., 2018** [100]	*n* = 120 (rIPC = 60) *n* = 60 (rIPC = 30)	Lower limb	3 cycles of 5 min with cuff inflation to 200 mmHg	↓ in 27% in SCr ↓ cystatin C, NGAL and SCr levels
**Candilio, L. et al., 2014** [101]	*n* = 178 (rIPC = 89)	Upper arm	2 cycles of 5 min with cuff inflation to 200 mmHg	↓ AKI incidence
**Venugopal, V. et al., 2010** [102] **Thielmann, M. et al., 2010** [103] **Savaj, S. et al., 2014** [104] **Zarbock, A. et al., 2015** [105] **Yamanaka, T. et al., 2015** [106] **Zarbock, A. et al., 2017** [107] **Stokfisz, K. et al. (2020) A** [108]	*n* = 78 (rIPC = 38) *n* = 56 (rIPC = 27) *n* = 96 (rIPC = 48) *n* = 240 (rIPC = 120) *n* = 94 (rIPC = 47) *n* = 240 (rIPC = 120) *n* = 28 (rIPC = 14)	Upper arm	3 cycles of 5 min with cuff inflation to 200 mmHg	↓ AKI incidence ↓SCr ↓ CI-AKI incidence ↓ 3-month incidence of major adverse kidney events
**Zagidullin, N. et al., 2016** [109]	*n* = 51 (rIPC = 26)	Upper arm	3 cycles of 5 min with cuff inflation 50 mmHg above systolic pressure	↓ CI-AKI incidence
**Meersch, M. et al., 2020** [110]	*n* = 180 (rIPC = 100)	Upper arm	3 cycles of 3, 7 or 10 min with cuff inflation of 200 mmHg	↑ TIMP-2 IGFBP7 ↓ AKI incidence
**Igarachi, G. et al., 2013** [111] **Moretti, C. et al., 2017** [112] **Kim, T.K. et al., 2017** [113] **Zhou, H. et al., 2019** [114]	*n* = 60 (rIPC = 30) *n* = 442 (rIPC = 223) *n* = 160 (rIPC = 80) *n* = 130 (rIPC = 65)	Upper arm	4 cycles of 5 min with cuff inflation to 200 mmHg	↓ CI-AKI incidence ↓ AKI incidence ↓ postoperative AKI
**Er, F. et al., 2012** [115] **Elserafy, A.S. et al., 2017** [116]	*n* = 82 (rIPC = 41) *n* = 100 (rIPC = 50)	Upper arm	4 cycles of 5 min with cuff inflation 50 mmHg above systolic pressure	↓ CI-AKI, ↓ SCr and sCystatin C
**NO RENOPROTECTION**				
**Walsh, S.R. et al., 2010** [117]	*n* = 40 (rIPC = 18)	Right and left iliac artery	10 min of ischemia in left and right common iliac artery	No protection
**Choi, Y.S. et al., 2011** [118]	*n* = 76 (rIPC = 38)	Lower limb	3 cycles of 10 min with cuff inflation to 250 mmHg	No difference in SCr, cystatin C and NGAL
**Chen, Y. et al., 2013** [119]	*n* = 60 (rIPC = 40)	Upper part of the leg	3 cycles of 5 min with cuff inflation to 300 mmHg	No difference in SCr, and NGAL
**Hu, Q. et al., 2015** [120]	*n* = 201 (rIPC = 101)	Right thigh	3 cycles of 5 min with cuff inflation to 600 mmHg after cross-clamping aorta	No statistical differences in BUN, SCr and cystatin C
**Pedersen, K.R. et al., 2012** [121]	*n* = 105 (rIPC = 54)	Lower limb	4 cycles of 5 min with cuff pressure 40 mmHg above systolic pressure	No renoprotection, and no difference in sNGAL, uNGAL, sCystatin C and SCr
**Rahman, I.A. et al., 2010** [122] **Young, P.J. et al., 2012** [123] **Pinaud, F. et al., 2016** [124] **Singh, G.B. et al., 2016** [125] **Bagheri, S. et al., 2018** [126] **Wojciechowska, M. et al., 2018** [127]	*n* = 162 (rIPC = 80) *n* = 96 (rIPC = 48) *n* = 99 (rIPC = 49) *n* = 102 (rIPC = 51) *n* = 177 (rIPC = 87) *n* = 124 (rIPC = 62)	Upper limb	3 cycles of 5 min with cuff inflation to 200 mmHg	No renal protection No difference in AKI incidence No effect CI-AKI incidence
**Murphy, N. et al., 2014** [128]	*n* = 62 (rIPC = 31)	Upper limb	3 cycles of 5 min with cuff inflation 100 mmHg above systolic pressure	No difference in AKI incidence
**Gallagher, S.M. et al., 2015** [129]	*n* = 86 (rIPC = 43)	Upper arm	3 cycles of 5 min with cuff inflation 50 mmHg above systolic pressure	No difference in AKI incidence
**Nouraei, S.M. et al., 2016** [130]	*n* = 99 (rIPC = 50)	Upper thigh	3 cycles of 5 min with cuff inflation 20 mmHg above systolic pressure	↓ in renal impairment
**Song, J.W. et al., 2018** [131]	*n* = 244 (rIPC = 120)	Upper arm	3 cycles of 5 min with cuff inflation to 250 mmHg	No difference in AKI incidence
**Hausenloy, D.J. et al., 2015** [132] **Stokfisz, K. et al., 2020 B** [133]	*n* = 1612 (rIPC = 801) *n* = 101 (rIPC = 50)	Upper arm	4 cycles of 5 min with cuff inflation to 200 mmHg	No difference in AKI incidence No difference in CI-AKI
**Menting, T.P. et al., 2015** [134]	*n* = 72 (rIPC = 36)	Upper arm	4 cycles of 5 min with cuff inflation to 10 mmHg below diastolic pressure	No difference in AKI incidence
**Valappil, S.P. et al., 2017** [135] **Ghaemian, A. et al., 2018** [136]	*n* = 100 (rIPC = 50) *n* = 132 (rIPC = 66)	Upper arm	4 cycles of 5 min with cuff inflation 50 mmHg above systolic pressure	No difference in CI-AKI incidence

SCr: serum creatinine, AKI: acute kidney injury, CI-AKI: contrast-induced acute kidney injury, CSA-AKI: cardiac surgery-associated acute kidney injury, NGAL = neutrophil gelatinase-associated lipocalin, BUN = blood urea nitrogen, TIMP-2 = tissue inhibitor of metalloproteinases 2, IGFBP7 = insulin-like growth factor binding protein 7.

## Data Availability

Not applicable.
